# Scoring the Icecap-A Capability Instrument. Estimation of a UK General Population Tariff†

**DOI:** 10.1002/hec.3014

**Published:** 2013-11-20

**Authors:** Terry N Flynn, Elisabeth Huynh, Tim J Peters, Hareth Al-Janabi, Sam Clemens, Alison Moody, Joanna Coast

**Affiliations:** a Centre for the Study of Choice (CenSoC), University of Technology SydneySydney, Australia; b School of Clinical Sciences, University of BristolBristol, UK; c Health Economics Unit, School of Health and Population Sciences, University of BirminghamBirmingham, UK; d National Centre for Social ResearchLondon, UK; e Institute of Epidemiology and Health Care, University College LondonLondon, UK

**Keywords:** discrete choice experiments, best–worst scaling, capability approach, well-being, economic evaluation, variance heterogeneity

## Abstract

This paper reports the results of a best–worst scaling (BWS) study to value the Investigating Choice Experiments Capability Measure for Adults (ICECAP-A), a new capability measure among adults, in a UK setting. A main effects plan plus its foldover was used to estimate weights for each of the four levels of all five attributes. The BWS study was administered to 413 randomly sampled individuals, together with sociodemographic and other questions. Scale-adjusted latent class analyses identified two preference and two (variance) scale classes. Ability to characterize preference and scale heterogeneity was limited, but data quality was good, and the final model exhibited a high pseudo-*r*-squared. After adjusting for heterogeneity, a population tariff was estimated. This showed that ‘attachment’ and ‘stability’ each account for around 22% of the space, and ‘autonomy’, ‘achievement’ and ‘enjoyment’ account for around 18% each. Across all attributes, greater value was placed on the difference between the lowest levels of capability than between the highest. This tariff will enable ICECAP-A to be used in economic evaluation both within the field of health and across public policy generally. © 2013 The Authors. *Health Economics* published by John Wiley & Sons Ltd.

## 1. Introduction

Among many practitioners of economic evaluation, there is a growing view that the measurement of health alone (for example through Quality-Adjusted Life-Years or QALYs) in economic evaluation is often insufficient (Oliver *et al*., 2002; Coast, 2004; Ryan *et al*., 2006). This is particularly the case where the interventions to be evaluated combine health and social care, where they are concerned with public health and where there are significant ‘spill-over’ effects of interventions—for example, impacts on carers or family, or on the wider general public. In all these situations, a broader measure of well-being is required.

One such approach to broader well-being is to draw on Sen's capability approach (Sen, 1992; Sen, 1993). This is being explored through a number of avenues within health economics (Anand and van Hees, 2006; Coast *et al*., 2008a; Lorgelly *et al*., 2010). One measure available is the Investigating Choice Experiments Capability Measure for Adults (ICECAP-A) (Al-Janabi *et al*., 2012), which draws on a capability approach in that it comprises a measure of a person's ability to achieve important ‘functionings’. ICECAP-A was developed using rigorous qualitative work. Initial in-depth qualitative interviews identified capabilities that are important to people in their lives, and semistructured interviews were then used to ensure that the language used is comprehensible. In brief, ICECAP-A has the following five attributes, each of which can take one of four levels ranging from full capability to no capability:

- Stability (being able to feel settled and secure)
- Attachment (being able to have love, friendship and support)
- Autonomy (being able to be independent)
- Achievement (being able to achieve and progress)
- Enjoyment (being able to have enjoyment and pleasure)

ICECAP-A is already being used in 16 clinical and other studies across the UK, USA, Australia and New Zealand, showing the high international demand for such a measure for economic evaluation. Moreover, an earlier similar measure for use with older people, ICECAP-O (Grewal *et al*., 2006; Coast *et al*., 2008b; Flynn *et al*., 2010), is already being used in 30 studies in countries including the UK (Flynn *et al*., 2011), USA, the Netherlands (Makai *et al*., 2012), Canada (Davis *et al*., 2012) and Australia (Couzner *et al*., 2012). These measures are particularly valuable where it is important to go beyond health and consider benefits in terms of a person's overall well-being.

Conceptual issues in valuing capabilities have been discussed previously (Cookson, 2005; Coast *et al*., 2008a). There are at least two practical issues in conducting valuation using ordinal tasks. First, there is a normative decision for those wishing to use a capability well-being measure in health economic evaluation, who must decide whether it should have QALY properties and specifically whether it should be anchored such that zero is equivalent to death. If that normative decision is that a measure should have such properties and ordinal tasks are to be used in valuation, it is important to recognize the implications of random utility theory (RUT), which underpins discrete choice models (Thurstone, 1927; McFadden, 1974). For researchers wanting values with QALY properties, treating ‘death’ as just another state in a ranking task (McCabe *et al*., 2006) is conceptually and theoretically flawed (Flynn *et al*., 2008). Two solutions have been proposed (Flynn, 2010a): the second-best one—using time trade-off tasks to rescale (relative) values so they are properly anchored to death (zero)—is being used in social care and adolescent health projects (Potoglou *et al*., 2011; Ratcliffe *et al*., 2011) whereas the ideal solution—that of including length of life as an attribute—was recently demonstrated (Bansback *et al*., 2012).

In terms of the normative decision, for the previous ICECAP measure, ICECAP-O, values are anchored to the ‘no capability’ state, which is the zero point on the scale; although death implies no capability, the reverse is not necessarily true. That anchoring decision reflects a normative decision (Coast *et al*., 2008b) based, in part, on the use of capabilities as a measure of (unmet) need for identifying those in particularly poor states (Flynn *et al*., 2011), rather than as a ‘super-QALY’ for use in comparing quantity and (broadly defined) quality of life. On the other hand, projects in social care and adolescent health have decided to retain the zero as the death anchor, to preserve QALY properties (Potoglou *et al*., 2011; Ratcliffe *et al*., 2011). Here the decision was made for ICECAP-A to retain the anchors as full capability and no capability, as for ICECAP-O. With the making of such a decision, it might be thought that the measurement issues described above are no longer relevant, but RUT issues are just as pertinent in the absence of QALY anchoring. Indeed, the more general issue underlying the ‘death state’ issue is that RUT assumes respondents make errors. Deterministic decisions or indeed any heterogeneity in choice consistency (respondent ‘certainty’ or ‘ability to do the choice task’) constitutes a violation of RUT and produces biased estimates, unlike the case for linear regression models (Yatchew and Griliches, 1985). The possibility of variance scale heterogeneity is finally being recognized; design strategies to avoid it (Flynn, 2010a) and methods to adjust for it when present (Fiebig *et al*., 2010; Flynn *et al*., 2010) are now available.

As indicated in earlier work, ‘the definitive process by which values should be elicited for capabilities is unresolved’ (Coast *et al*., 2008b), and a number of approaches have been tried including linking with life satisfaction (Anand *et al*., 2009). Previous work with ICECAP has used ‘Cookson's compromise’ in assuming that population values obtained from choice-based tasks can be used as evidence for valuation with the capability approach (Cookson, 2005). Discrete choice-based valuation tasks are increasingly used, both within and outwith a capability approach, because ordinal tasks make weaker assumptions about human decision-making processes than cardinal tasks. Examples include ranking studies (McCabe *et al*., 2006), discrete choice experiments (DCEs) (Ryan *et al*., 2006) and profile case (case 2) best–worst scaling (BWS) studies (Coast *et al*., 2008b; Potoglou *et al*., 2011).

This paper reports the process by which these capability values have been obtained for ICECAP-A and the resulting tariff for use in economic evaluation. It also briefly discusses how the new measure, with its associated values, can be used to aid the decision-making process in health care.

## 2. Methods

### 2.1. Valuation study task

The particular type of BWS valuation task that was conducted is known as the profile case or case 2 (Flynn *et al*., 2007; Flynn, 2010a). In this exercise, respondents are presented with a set of hypothetical scenarios. Each scenario provides a profile containing all of the attributes, with each attribute at a particular level; for example, a profile might include the highest levels of autonomy and achievement, the middle level of stability and the lower levels of attachment and enjoyment. Respondents are asked to choose from within each profile which attribute is best and which is worst—here, for example, they might choose a high level of achievement as the best thing within the profile and the low level of attachment as the worst. The estimated capability values are then a function of choice frequencies (Thurstone, 1927; McFadden, 1974); how often a respondent chooses level X of capability attribute A or level Y of capability attribute B provides an indication of how much the respondent values level X of A over level Y of B. As such, the econometric model is a conditional logit in which choice options are simply attribute levels rather than complete (capability) states. The main difference from a standard DCE (based on first choices) is that ‘worst’ choice data are appended to ‘best’ choice data with all independent variables taking a sign change (to reflect the fact that ‘worst’ from options with utilities 2, 4, 7, 8 is observationally equivalent to ‘best’ from options with utilities −2, −4, −7, −8).

### 2.2. Experimental design

Given five attributes, each with four levels, for the ICECAP-A, it was not feasible to provide respondents with all 1024 (4^5^) possible scenarios, and in any case, many of the states that are required to estimate two-way and higher-order interactions cannot be administered in Profile Case BWS (Flynn, 2010b). Therefore, the number was reduced using an orthogonal main effects plan (OMEP) to give a set of scenarios where all attributes are statistically independent. This enables independent estimation of the values that people associate with each level of every attribute, assuming no interactions between them. The OMEP in 16 states was obtained from an online catalogue (http://www2.research.att.com/~njas/oadir/ and design oa.16.5.4.2). This gives a design showing how attributes and levels should appear relative to one another but does not dictate which attribute levels are which within the design. For example, the design might specify level 1 of attribute 1 and level 4 of attribute 2, but level 1 does not have to be assigned to the top level, nor level 4 to the bottom. Therefore, all possible coding schemes were tested in Microsoft Excel to minimize the number of profiles in which either best or worst was too easy (e.g. if four attributes appear with their worst level and one appears with its best level, it is ‘easy’ to choose which attribute is best). In practice this meant that, typically, the respondent had to choose best from two attributes that were each presented at a high level and choose worst from two that were each presented at a low level. The OMEP was used for half the respondents, and its ‘foldover’ or mirror image was used with the other half. This increases the number of scenarios relative to the number of parameters being estimated and follows practice from other studies (Coast *et al*., 2008b). Individuals were randomly allocated to either the original OMEP or the foldover (see Appendix 1 in the Supporting Information).

### 2.3. Overall survey design and conduct

The valuation exercise was accompanied by a number of sociodemographic items, attitudinal questions and outcome measures. The survey was interview-administered by National Centre for Social Research (NatCen) interviewers using computer-assisted personal interviewing, thus allowing face-to-face discussion of any queries relating to the survey. Ethics approval was obtained from University of Birmingham Life and Health Sciences Ethical Review Committee, approval number ERN_08-093.

#### 2.3.1. Piloting

The survey was piloted in May 2010 in a convenience sample of 28 individuals from the South-East of England. Following piloting, small changes were made to the content of the survey and instructions, but participants appeared to be able to complete the valuation task reasonably easily, and there were no major changes prior to the main survey.

#### 2.3.2. Sampling

To determine the appropriate sample size for a valuation exercise, prior knowledge about the variability in preferences for the attributes of the measure would be needed. Unfortunately, this was not known. Information from a previous study of a different, but similar, measure was therefore used (Coast *et al*., 2008b), which suggested that a sample of 400 complete responses would be sufficient to estimate a set of index values for the measure that could be used for the population, and could enable some investigation of preference and scale heterogeneity (Flynn *et al*., 2010).

A representative sample of British adults was approached to take part in the valuation exercise. Addresses were randomly selected for the survey from the Postcode Address File in Great Britain using a two-stage stratified design (stratified for geographic area and socioeconomic deprivation). At each selected address, one adult was randomly selected to take part in the survey. Each household received a postal invitation to participate, which the designated interviewer followed up in person. There were up to six attempts to make contact and elicit either an agreement to participate or a refusal. Anticipating a response in the region of 50% (again based on prior experience), NatCen approached 887 addresses.

### 2.4. Analysis

#### 2.4.1. Choice data summary

First, a table of all 320 possible best–worst pairs of attribute levels was constructed, showing the frequency with which attributes were chosen. This provides a preliminary indication of which pairs were chosen frequently and, by summing to the margins, provides a model-free set of best (row) and worst (column) estimates of the capability scores for all 4 × 5 = 20 attribute levels.

#### 2.4.2. Best-minus-worst data

**Figure 1:**
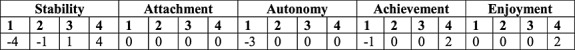
Hypothetical set of scores for an individual: This respondent cares most about stability. It should be noted that a property of the design is that no other attribute can also have a −4 or +4 for an attribute level. The respondent either ignored attachment or picked some/all levels the same number of times as best as worst. She dislikes level 1 of autonomy (no independence) but appears to care little about the upper levels, unlike enjoyment, for which it is only the top level that she cares about. Achievement shows an intuitive nondecreasing set of scores across increasing levels

A second set of analyses was conducted using the best-minus-worst scores (Marley and Louviere, 2005; Flynn, 2010a). Within the OMEP design, each attribute level appeared on four occasions within the 16 scenarios. It could therefore have been picked as best up to four times and as worst up to four times for each person. These best-minus-worst scores are calculated for each attribute by determining the number of times that a person picked an attribute level (for example, ‘I am able to be completely independent’) as best and subtracting from that the number of times that they picked it as worst. Scores can therefore range from −4 (never picked as best and always picked as worst) to +4 (never picked as worst and always as best). Scores for respondents provide an immediate indication of which attribute levels they value. Figure 1 gives an example of the scores for one individual.

It is important to adjust for heterogeneity in both preference and variance scale at the level of individual respondents (Louviere *et al*., 2000; Swait and Adamowicz, 2001; Louviere *et al*., 2002; Fiebig *et al*., 2010). Variance scale is concerned with how consistent individuals are in making their choices: some individuals are more consistent and others are less so. If this is not adjusted for, people may be thought to have different preferences where, in fact, their preferences are similar but they are just less consistent in making them. Although this makes the analysis considerably more complex, it is vital in estimating a set of population values (Flynn *et al*., 2010), because not accounting for this sort of heterogeneity leads to bias in the mean estimates obtained from limited dependent variable (such as probit and logit) models (Yatchew and Griliches, 1985). A series of cluster analyses based on functions of the best-minus-worst scores was conducted, with further details provided in Appendix 2 (Supporting Information). These did not provide the final capability scores but were essential in ensuring that the main scale-adjusted latent class analyses (SALCs) did not give spurious solutions, such as finishing at a local maximum of the likelihood function.

#### 2.4.3. Scale-adjusted latent class estimates and a UK tariff

The clustering analyses, by being based on continuous outcomes (probabilities), were anticipated to be more powerful in identifying patterns of heterogeneity; as such, they were crucial in helping inform heterogeneity analyses in the SALCs. Only the latter provide the key latent capability scores. Therefore, a series of SALCs was conducted to identify preference and scale heterogeneity in the choice data using Latent Gold Choice 4.5 (with syntax module) software (Flynn *et al*., 2010). The behavioural model is assumed to be a logit model, and the preference distribution is a discrete finite mixture of logit models assumed to comprise latent classes of respondents with the same preference part-worth utilities and/or scale. Whereas traditional latent class models (Greene and Hensher, 2003) can potentially confound heterogeneity in preferences and individual differences in error variance, scale-adjusted latent class choice models separately consider both types of response heterogeneity allowing for respondents to differ in their level of choice uncertainty (Flynn *et al*., 2010).

The estimated SALC models used the five sums of squares variables (one for each attribute) from the cluster analyses in Appendix 2 (Supporting Information) as predictors of preference class membership and the Empirical Scale Parameter as a predictor of scale class. These predictors were used to help stabilize the solutions (Flynn *et al*., 2010). The Bayesian Information Criterion was used to help guide model selection, but stability of solutions was also used to decide on the optimal model. As is the case with maximum likelihood estimation, the optimization algorithm (k-means) does not necessarily guarantee that a set of parameter values uniquely maximizes the log-likelihood. Thus, different starting seeds for the algorithm should lead to the same solution to ensure with some confidence that a global maximum has been found.

Once the optimal model was identified, sociodemographic variables were tested for significance in predicting preference and/or scale class membership. This allows any preference heterogeneity to be better characterized and modelled. Univariable analyses were conducted to test for these possible predictors before a multivariable analysis was performed (Peters *et al*., 2003; Patel *et al*., 2005).

Finally, the heterogeneity-adjusted population level tariff was calculated in the following way:

- The average values across all respondents were calculated, by taking the mean of the sets of preference class estimates, weighted by the average mixing coefficients across the sample. These naturally account for preference class membership and ‘net out’ differences in scale.
- A linear transformation was applied to the final set of 20 estimates to produce a tariff anchored at zero for the state of ‘no capability’ (11111) and a value of one for the state of ‘full capability’ (44444) (Coast *et al*., 2008b).

## 3. Results

From the initial sample of addresses selected, 805 were eligible (that is, were both residential and occupied). From these, 422 interviews resulted (52% response rate). Among the nonresponders, 227 (34%) refused to participate, 60 (7%) were not contactable and 44 (5%) were contacts that were unproductive for some other reason (for example, the potential respondent being too ill to take part, or being unable to communicate in English). Of the 422 interviews, 418 (99%) were completed, and 413 (98%) resulted in full choice data for the task. Analyses are reported for the 413 respondents who provided complete choice data. Table I shows the characteristics of survey respondents.

**Table I:** Descriptive statistics (n = 413)

Variable	Categories	Frequency	Percent
Age	18–34	92	22.4
	35–54	136	33.1
	55–74	134	32.6
	75+	49	11.9
Ethnicity	White	369	91.1
	Nonwhite	36	8.9
	Total	405	100
Country	England	341	82.6
	Wales/Scotland	72	17.4
Marital status	Never married	93	22.7
	Widowed	50	12.2
	Divorced	73	17.9
	Married/Civil partner	193	47.2
Number of adults	1	143	35.0
	2	207	50.6
	3+	59	14.4
Number of children	0	300	73.4
	1	43	10.5
	2+	66	16.1
Income tertiles	Lowest tertile	132	32.0
	Middle tertile	98	23.7
	Highest tertile	123	29.8
	Don't know/refused	60	14.5
Gender	Male	156	37.8
	Female	257	62.2
Any qualification	Yes	280	68.3
	No	130	31.7

### 3.1. Choice data summary

Table II provides non-model-based estimates of the best and worst values (summing to the margins of rows and columns respectively).

**Table II:** Best–worst pair frequencies (n = 413). Row (column) means represent the average number of times an attribute level was picked as best (worst) across all states

		Stability				Attachment				Autonomy				Achievement				Enjoyment				Best mean
	Level	1	2	3	4	1	2	3	4	1	2	3	4	1	2	3	4	1	2	3	4	
Stability	1					10	1	0	1	4	3	1	0	3	3	3	0	5	3	4	0	2.6
	2					73	13	2	2	32	8	6	2	17	17	1	5	19	16	4	2	13.7
	3					171	44	2	3	74	45	9	6	80	67	4	5	123	48	13	1	43.4
	4					156	49	30	2	121	115	38	7	82	21	23	1	97	115	11	12	**55.0**
Attachment	1	2	0	1	0					4	2	1	2	1	0	0	1	0	1	1	1	1.1
	2	57	17	6	2					25	6	3	2	14	7	2	2	71	20	3	2	14.9
	3	88	41	3	7					120	78	20	2	113	54	15	8	85	64	3	17	44.9
	4	150	83	11	6					84	88	20	2	81	114	10	10	150	101	19	3	**58.3**
Autonomy	1	1	2	1	0	2	0	0	1					2	2	0	0	1	1	0	1	0.9
	2	23	7	0	1	13	4	1	1					14	4	2	2	27	8	3	2	7.0
	3	66	40	3	3	93	26	8	0					33	15	7	4	47	53	2	3	25.2
	4	72	36	9	3	76	103	1	1					27	28	3	7	73	70	13	4	**32.9**
Achievement	1	3	0	0	1	2	1	1	0	2	0	1	0					2	1	1	0	0.9
	2	35	3	2	0	19	10	1	0	10	1	5	2					31	6	2	2	8.1
	3	64	23	2	1	116	31	3	1	52	4	8	4					51	33	2	0	24.7
	4	51	52	7	5	72	48	6	1	31	30	4	4					32	20	5	2	23.1
Enjoyment	1	0	0	0	1	4	1	1	2	1	0	0	0	0	1	0	0					0.7
	2	10	3	1	1	21	2	2	0	9	2	0	0	4	2	0	2					3.7
	3	68	14	4	2	95	21	1	1	76	30	1	5	13	15	1	0					21.7
	4	58	51	5	1	89	31	11	3	45	21	12	4	21	29	7	1					24.3
Worst mean		**46.8**	23.3	3.4	2.1	**63.3**	24.1	4.4	1.2	43.1	27.1	8.1	2.6	31.6	23.7	4.9	3	**50.9**	35	5.4	3.3	

Values represent the number of times that the best (row) and worst (column) pair was picked. The three most liked attribute levels and the three most disliked ones are in bold.

The ‘best’ data indicate very strong relative preferences for stability and attachment, with autonomy being the third most important attribute. The ‘worst’ data suggest strong aversion to low levels of attachment and enjoyment. Achievement does not appear to have a large impact upon best or worst preferences. These results suggest that attachment and stability have a relatively large span of the ‘capability space’ whereas autonomy has a moderate span and enjoyment only has impact if it is totally absent in life. Appendix 2 (Supporting Information) details results of cluster analyses of the best-minus-worst data to understand how preference and variance scale heterogeneity manifested themselves.

### 3.2. Scale-adjusted latent class estimates and a UK tariff

Three scale class solutions were unstable for some starting value seeds (and were considered to be unlikely given the number of modes in Figure 1), as were two-scale three- and four-preference class solutions; hence, a two-scale two-preference class solution was preferred. (Summary statistics for the various SALC models are available from the authors on request.) The coefficients for each class are provided in Table III. This model was characterized by strongest preferences for the following:

1. Attachment (n = 216)
2. Stability, with autonomy being a close second (n = 197)

**Table III:** Scale-adjusted latent class analysis final model coefficient estimates and tariff

		Latent class 1		Latent class 2		Tariff
		Coef	S.E.^a^	Coef	S.E.^b^	
Stability (mean)^b^		0.114	0.017	0.125	0.016	–
Attachment (mean)		0.140	0.019	−0.013	0.012	–
Autonomy (mean)		−0.093	0.012	−0.007	0.014	–
Achievement (mean)		−0.042	0.012	0.017	0.012	–
Enjoyment (mean)		−0.120	0.014	−0.122	0.014	–
Stability (4)^c^		0.735	0.049	0.872	0.053	0.2221
Stability (3)		0.490	0.039	0.592	0.037	0.1915
Stability (2)		−0.197	0.029	−0.272	0.035	0.1013
Stability (1)		−1.028	0.068	−1.193	0.072	−0.0008
Attachment (4)		1.023	0.055	0.769	0.053	0.2276
Attachment (3)		0.644	0.039	0.493	0.036	0.1890
Attachment (2)		−0.253	0.037	−0.187	0.032	0.0964
Attachment (1)		−1.413	0.076	−1.075	0.073	−0.0239
Autonomy (4)		0.468	0.040	0.912	0.061	0.1881
Autonomy (3)		0.314	0.030	0.507	0.036	0.1560
Autonomy (2)		−0.137	0.022	−0.296	0.034	0.0836
Autonomy (1)		−0.646	0.050	−1.123	0.071	0.0063
Achievement (4)		0.467	0.045	0.710	0.052	0.1811
Achievement (3)		0.308	0.032	0.484	0.035	0.1588
Achievement (2)		−0.150	0.025	−0.230	0.031	0.0909
Achievement (1)		−0.625	0.060	−0.963	0.069	0.0210
Enjoyment (4)		0.684	0.042	0.697	0.046	0.1811
Enjoyment (3)		0.434	0.035	0.485	0.035	0.1540
Enjoyment (2)		−0.264	0.031	−0.269	0.037	0.0693
Enjoyment (1)		−0.854	0.055	−0.913	0.061	−0.0026
	Coef^d^	S.E.				
Est' scale factor (1)	1	–				
Est' scale factor Scale (2)	3.543	0.138				

a Robust standard errors to account for clustering at respondent level.

b Attribute impact, i.e. average capability score across the attribute's four levels. Estimates are effect coded.

c Effect-coded capability scores for levels (measuring deviation from attribute impact). Total capability score for an attribute level calculated as sum of relevant attribute impact and level score.

d Indicating the multiplicative factor to apply to the preference class coefficient estimates for each scale class. The vast majority of respondents were in scale class 2.

There appear to be very small variances on the latent scale, at least for those 351 respondents in the high scale (low variance) class. This is evident from the high pseudo-*r*-squared for this model (0.34) and the large differences between levels for certain attributes (stability and attachment in particular): multiplying the ‘raw’ coefficient estimates by the relative scale factor (3.543) gives estimates for respondents in the high scale (low variance) class, and these produce highly skewed choice probabilities when inserted into the logit function.

Univariable associations between the class membership solution reported above and (i) sociodemographic and (ii) five own capability responses were tested in Latent Gold Choice 4.5 (+syntax) and Stata (11MP). Results from the logistic regression analyses including Wald and likelihood ratio chi-squared test statistics were used to test for associations between the tested variable and the outcome. Chi-squared tests of significance showed an association at *p* < 0.05 for the following:

- Respondents who were married or in a civil partnership were more likely than those with other relationship status to value attachment. The probability of being in the class that particularly valued attachment decreased across the categories widowed, divorced and never married.
- People living in a two-person household had the strongest preference for attachment. Those living alone or with 2+ other people were more likely to place their highest values on stability (and autonomy).

In terms of scale class, increasing age was strongly associated with smaller scale, as was being in the lowest income tertile and not having a qualification.

Multivariable logistic regression produced no associations between any of the sociodemographic variables and preference class at the 5% level, and only income was associated with scale class (results not shown). In terms of own capability responses, attachment was the only attribute associated with differences in preferences. Increasing loneliness was associated with an increased focus on stability and autonomy at the expense of attachment: because these are cross-sectional data, the direction of causation between preferences and own capability is unknown. This remained in a multivariable logistic regression involving all five capability variables. For attachment, stability and autonomy lower capabilities are associated with lower scale factor (choice consistency). None of these remained significant at the 5% level in a multivariable logistic regression, although attachment and autonomy were significant at the 10% level.

Importantly, Table III also provides the final tariff for use in economic evaluations incorporating ICECAP-A as the measure of outcome. The tariff indicates that all five attributes make a significant contribution to an individual's capability well-being, with ‘attachment’ and ‘stability’ accounting for around 22% of the space each and ‘autonomy’, ‘achievement’ and ‘enjoyment’ accounting for around 18% of the space each. Across all attributes, greater value was placed on the difference between the lowest levels of capability than between the highest (that is, the difference in value between being able to achieve in a few aspects of life and no aspects of life was greater than that between being able to achieve in all aspects in life and many). The tariff is additive, and so, for example, an improvement in capability from a poorer state of 21231 to an improved state of 43342 would imply a shift in value from (0.1013 + [−0.0239] + 0.0836 + 0.1588 + [−0.0026]), equal to 0.3172, to (0.2221 + 0.1890 + 0.1560 + 0.1811 + 0.0693), equal to 0.8175.

## 4. Discussion

This is the first paper to report a population level tariff for the ICECAP-A capability measure for use in economic evaluation. Both the raw data and the final tariff suggest that all five attributes are important to people but that, at a population level, the relative preferences for stability and attachment are slightly stronger than those for autonomy, achievement and enjoyment. Further, the tariff suggests that people would place greater value on improving the capability of those with very low levels of capability than on improving the capability of those who already have a relatively high level of capability. The paper is the first to report a population level tariff from a DCE-type study that explicitly adjusts for heterogeneity in both preferences and variance scale at the level of the individual respondent and thus provides unbiased estimates of population value. Other similar studies have thus far not made such explicit adjustments to the tariffs reported or have done so only in follow-up analyses (Flynn *et al*., 2010; Potoglou *et al*., 2011).

The task that people were asked to complete was feasible, with the vast majority of respondents being able to provide complete choice data; the fact that there were no respondents with an empirical scale factor of over 6 suggests that nobody obviously misunderstood the nature of the best–worst task (nor tried to game the system). The approach to sampling meant that individuals had an equal chance of being selected for the survey, although there were higher responses from some groups, with the result that females and older persons are slightly overrepresented compared to the UK population. The rigorous analytical processes detailed in the online appendices (Supporting Information) were imperative because of the unique and critical problem in making inferences from all such studies. The confounding between means and variance on the latent (in this case, capability) scale is perfect, and there are technically an infinite number of possible solutions. Reducing this to a manageable number required the use of theory, heuristic reasoning and the ability to recognize what patterns of choice behaviour are consistent with particular psychological and economic theories. Although all choice tasks of this kind make assumptions when conducting heterogeneity analyses, those made here were relatively weak: no distributional assumptions were made about preferences in the wider population, and the assumption that scale heterogeneity manifested itself as a simple ‘cognitive impairment/education’ effect, leading some people to be more consistent than others across all their choices, is not unreasonable.

There were, however, also some limitations in the analysis. The parsimony and lack of distributional assumptions (unlike the popular mixed-logit model) in the SALCs were probably responsible (at least in part) for certain problems in their implementation. First, they had problems decomposing preference and scale without the use of additional variables known to be associated with either of these. Second, when such decomposition was successful, most of the findings from the cluster analyses could not be replicated in the SALC modelling at conventional levels of statistical significance. Given the broad agreement between the results from the SALC model and the cluster analysis (which was more powerful by working directly on the latent capability scale), this suggests that, though producing reasonable results, the SALC may have been underpowered.

A second limitation was the inability to estimate interactions, an inevitable consequence of using Case 2 BWS over a conventional DCE. However, this likely made the results more representative of the general population: the ICECAP-O valuation exercise showed that a conventional DCE task was associated with higher cognitive burden and larger amounts of essentially useless data (Flynn *et al*., 2013). Additionally, a DCE capable of estimating even only two-way interactions would have been prohibitively costly, in terms of sample size. Work underway to value the ICECAP-Supportive Care Measure (ICECAP-SCM) for use at the end of life (Sutton and Coast, 2013) is incorporating both types of choice method to draw upon their respective advantages.

For the first time, this study provides a generic measure of well-being that can be used across health and other areas of public provision of goods and services. The measure focuses on what is ultimately important to people in their lives—their overall well-being. Two examples may serve to show why this is important. First, assume an intervention to reduce alcohol consumption. A standard QALY-type analysis would compare the change in health among those receiving the intervention and any changes in health among those not receiving the intervention. It would not capture improvements in, for example, increased security/stability among those living in areas affected by increased alcohol consumption. The standard QALY analysis might underestimate the impact of the intervention whereas the ICECAP-A measure with its associated values could capture both impacts using the same measure. Second, assume a comparison of an intervention that improves an older person's heath minimally with the provision of a wheelchair that does not improve the person's health but provides the older person with a much greater independence of mobility. With the standard QALY analysis, such a comparison is difficult to make, but with ICECAP-A, the impacts of both interventions on the person's well-being could be captured.

The measure does not provide a QALY, being a measure of capability that is anchored at no capability and full capability, but it is possible to account for death: in terms of capability, a person who has died has no capability, and their loss of well-being can be captured in this way (of course, this does not mean that the reverse is true). The measure can also be adjusted for time, with analyses estimating gains in years of full capability equivalence. Thus, although not a QALY (because the measure is not anchored on death and thus not adjusting life years for quality), it is able to be used in a similar way to QALYs because ‘no capability’ provides a meaningful lower anchor. Inferences from such studies will help institutions such as the National Institute for Health and Care Excellence in the UK to better understand the health and wider nonhealth implications of new technologies and public health policies. For this sort of estimation, a robust tariff, as reported here, is vital.

This work provides a robust tariff for the UK. Where the measure is applied in other, non-UK, settings, it would be wise to estimate tariffs that appropriately represent the cultural values in these settings. Were this to be done, such studies should aim to recruit larger sample sizes, if at all possible, to avoid problems with the latent class analyses. Future valuation studies should be (much) larger and should adjust the sampling criteria to investigate, and if necessary adjust for, the associations found in the clustering analyses here. The suggestion that differences in preferences are associated with differences in own capability is further evidence that future studies must oversample citizens in poor capability states (and reweight at the analysis stage) to guard against the possibility that there are unobserved latent classes present in these data (Flynn, 2010a; Flynn, 2010b).

Future research is also needed to see how well the tariff performs in practice and, in particular, to investigate the sensitivity to change of the measure in clinical trials and decision analytic studies, to ensure the following: (i) it is sensitive to change in health across a range of interventions and conditions, and (ii) it does indeed capture change across a range of nonhealth consequences of interventions.

To conclude, this paper has drawn on an alternative extra-welfarist approach to evaluation, Sen's capability approach, and has provided a rigorously derived set of UK tariffs to use with the ICECAP-A capability well-being measure. These valuations, in conjunction with the ICECAP-A descriptive system, will, for the first time, enable researchers to conduct a capability-focused economic evaluation that can account for factors outside of health.

## Conflict of Interest

The authors are not aware of any financial or personal relationships between themselves and others that might be perceived as biasing the work reported here.
