# Supplementary material for: Scoring the Icecap-A Capability Instrument. Estimation of a UK General Population Tariff
Source: Health Econ. 2013 Nov 20;24(3):258–69. doi: 10.1002/hec.3014 (PMC4322472; doi:10.1002/hec.3014)
Supplement: Supplementary file 1 [file hec0024-0258-sd1.docx]

Appendix 1: ICECAP-A design matrix

| Scenario | Stability | Attachment | Autonomy | Achievement | Enjoyment |
| --- | --- | --- | --- | --- | --- |
| 1 | 2 | 1 | 1 | 2 | 3 |
| 2 | 1 | 1 | 4 | 3 | 4 |
| 3 | 4 | 3 | 4 | 2 | 2 |
| 4 | 1 | 4 | 1 | 4 | 2 |
| 5 | 1 | 3 | 3 | 1 | 3 |
| 6 | 4 | 1 | 3 | 4 | 1 |
| 7 | 2 | 4 | 4 | 1 | 1 |
| 8 | 3 | 1 | 2 | 1 | 2 |
| 9 | 1 | 2 | 2 | 2 | 1 |
| 10 | 2 | 3 | 2 | 4 | 4 |
| 11 | 3 | 4 | 3 | 2 | 4 |
| 12 | 3 | 3 | 1 | 3 | 1 |
| 13 | 2 | 2 | 3 | 3 | 2 |
| 14 | 4 | 4 | 2 | 3 | 3 |
| 15 | 3 | 2 | 4 | 4 | 3 |
| 16 | 4 | 2 | 1 | 1 | 4 |

Appendix 2: Preliminary cluster analyses

Empirical Scale Parameter (ESP)

The best-minus-worst scores were used to gain an initial understanding of the extent of preference and scale heterogeneity. In particular, scores were normalised (i.e. divided by 4), squared, and summed. For the five attributes, each of these sums of square (SS) was obtained. An overall value, summed across all attributes, was also calculated. This latter statistic is hereafter called the empirical scale parameter (ESP) and it shows the extent to which people are consistent in their choices ([Swait and Louviere, 1993](#_ENREF_28)). At one extreme, an ESP of zero indicates that values are spread randomly and suggests that the individual is likely to have made completely random choices. At the other extreme would be an ESP of 8. This would indicate a respondent who always chose one attribute as best, regardless of level, and similarly always chose a second attribute as worst, regardless of level. This would suggest a misunderstanding of the task (or a desire to move on to something else more quickly). A score of around four seems to be common where respondents have understood the task and have fairly consistent preferences. ESPs were calculated and plotted.

Cluster model estimates

Methods which identify heterogeneity independently of covariates are also useful. Latent class clustering analyses were conducted in Latent Gold Choice 4.5 (with syntax module) software, to identify clusters – “types” of respondent who differ in terms of their relative preferences for the five attributes. These models use maximum likelihood estimates to classify clusters based upon their posterior probability of class membership ([Hagenaars and McCutcheon, 2002](#_ENREF_17)). Findings from previous work of a similar type suggests the existence of a subgroup of respondents with either weak preferences or a low scale factor ([Flynn et al., 2010](#_ENREF_15)), and that one cluster would comprise respondents for whom the estimates were small in value or having a small ESP (around 2, indicating strong preference for only one attribute or a low scale factor). Therefore the number of clusters was increased until a cluster with a relatively small ESP was identified in these data.

The ESP distribution is provided in Figure 2. All values are below 6, suggesting that there were no respondents who tried to game the system or who obviously misunderstood the nature of the task. Interestingly, individuals whose own levels of capability were lower, were associated with lower ESP (results available from authors).

<Figure A1 here>

Table A21 contains means of SS1 through SS5 against categories of sociodemographic variables to provide a preliminary indication of sources of preference (SS) heterogeneity. There are a number of notable findings:

- Compared with females, males have a slightly stronger preference for enjoyment at the expense of autonomy
- Higher income is associated with a switch from autonomy towards attachment
- Increasing age is strongly associated with stronger preference for autonomy, at the expense of attachment and achievement
- Widowed and divorced individuals have relatively strong preferences for autonomy, at the expense of stability (divorcees) and achievement (widowed respondents)
- Having children is associated with weaker preferences for autonomy and enjoyment but stronger preferences for attachment

<Table A21 here>

Cluster model estimates

Summary statistics and mean SS values for the various cluster models are given in Table A22. While the Bayes Information Criterion (BIC) and Log Likelihood values continued to improve up until the 7-cluster model (results available from authors), the 3-cluster model (with pro-stability, pro-attachment and pro-autonomy clusters) was considered optimal. This was because the fourth cluster identified was a “low scale” (high variance) cluster: this was clear both from the fact it was drawn from all three existing clusters and its membership was strongly predicted by having a low ESP. These results are in accord with those in Table 3.

<Table A22 here>

Figure A1 – ESP distribution

Table A21 – SS across socio demographics

|  |  | Mean of SS/ESP* | | | | |
| --- | --- | --- | --- | --- | --- | --- |
| Variable | | Stability | Attachment | Autonomy | Achievement | Enjoyment |
| age | |  |  |  |  |  |
|  | 18-34 | 0.239 | 0.334 | 0.146 | 0.132 | 0.149 |
|  | 35-54 | 0.245 | 0.294 | 0.163 | 0.135 | 0.162 |
|  | 55-74 | 0.244 | 0.278 | 0.184 | 0.102 | 0.192 |
|  | 75+ | 0.187 | 0.316 | 0.227 | 0.094 | 0.177 |
| Ethnicity | |  |  |  |  |  |
|  | white | 0.237 | 0.301 | 0.175 | 0.115 | 0.173 |
|  | non-white | 0.241 | 0.282 | 0.183 | 0.179 | 0.115 |
| Country | |  |  |  |  |  |
|  | England | 0.240 | 0.297 | 0.174 | 0.118 | 0.170 |
|  | Wales/Scotland | 0.213 | 0.316 | 0.173 | 0.122 | 0.176 |
| Marital status | |  |  |  |  |  |
|  | Never married | 0.231 | 0.298 | 0.182 | 0.136 | 0.153 |
|  | Widowed | 0.241 | 0.291 | 0.220 | 0.096 | 0.152 |
|  | Divorced | 0.218 | 0.259 | 0.222 | 0.118 | 0.184 |
|  | Married/Civil partner | 0.244 | 0.321 | 0.140 | 0.119 | 0.176 |
| Number of adults | |  |  |  |  |  |
|  | 1 | 0.223 | 0.280 | 0.219 | 0.117 | 0.161 |
|  | 2 | 0.233 | 0.323 | 0.145 | 0.124 | 0.175 |
|  | 3+ | 0.279 | 0.272 | 0.165 | 0.113 | 0.171 |
| Number of children | |  |  |  |  |  |
|  | 0 | 0.235 | 0.290 | 0.183 | 0.115 | 0.177 |
|  | 1 | 0.278 | 0.299 | 0.144 | 0.145 | 0.134 |
|  | 2+ | 0.216 | 0.350 | 0.153 | 0.124 | 0.157 |
| Income tertiles | |  |  |  |  |  |
|  | lowest tertile | 0.229 | 0.281 | 0.201 | 0.119 | 0.170 |
|  | middle tertile | 0.249 | 0.307 | 0.173 | 0.106 | 0.165 |
|  | highest tertile | 0.251 | 0.332 | 0.138 | 0.126 | 0.154 |
|  | Don't know/refused | 0.196 | 0.271 | 0.187 | 0.124 | 0.221 |
| Gender | |  |  |  |  |  |
|  | Male | 0.219 | 0.301 | 0.159 | 0.122 | 0.199 |
|  | Female | 0.246 | 0.301 | 0.183 | 0.117 | 0.154 |
| Any qualification | |  |  |  |  |  |
|  | Yes | 0.249 | 0.301 | 0.164 | 0.127 | 0.159 |
|  | No | 0.210 | 0.298 | 0.198 | 0.104 | 0.191 |

*Individual SS values are scaled by their ESP values and averaged across all respondents.

Table A22 - Cluster analysis probability means

|  |  | **SS1** | **SS2** | **SS3** | **SS4** | **SS5** | **Membership Probability** | **Cluster Size** |
| --- | --- | --- | --- | --- | --- | --- | --- | --- |
| **3 Cluster Model** | |  |  |  |  |  |  |  |
|  | Cluster1 | 1.150 | 1.993 | 0.499 | 0.267 | 0.613 | 0.379 | 158 |
|  | Cluster2 | 0.669 | 0.777 | 1.280 | 0.446 | 0.511 | 0.335 | 139 |
|  | Cluster3 | 1.198 | 0.903 | 0.313 | 0.817 | 1.024 | 0.286 | 116 |
| **4 Cluster Model** | |  |  |  |  |  |  |  |
|  | Cluster1 | 1.391 | 0.978 | 0.283 | 0.620 | 1.014 | 0.324 | 135 |
|  | Cluster2 | 1.021 | 0.806 | 1.524 | 0.294 | 0.655 | 0.240 | 94 |
|  | Cluster3 | 0.847 | 2.459 | 0.436 | 0.271 | 0.594 | 0.238 | 100 |
|  | Cluster4 | 0.532 | 0.897 | 0.741 | 0.748 | 0.351 | 0.198 | 84 |
